# *PeCIN8* expression correlates with flower size and resistance to yellow leaf disease in *Phalaenopsis* orchids

**DOI:** 10.1186/s12870-023-04567-3

**Published:** 2023-11-07

**Authors:** Yan-Jeng Wu, Shu-Yun Chen, Fu-Cheng Hsu, Wen-Luan Wu, Ting-Fang Hsieh, Jiunn-Feng Su, Yung-Hsiang Lai, Pen-Chih Lai, Wen-Huei Chen, Hong-Hwa Chen

**Affiliations:** 1 Department of Life Sciences, National Cheng Kung University, Tainan, 701 Taiwan; 2 Present Address: Department of Agronomy, National Chung-Hsin University, Taichung, Taiwan; 3 Plant Pathology Division, Taiwan Agricultural Research Institute, Ministry of Agriculture, Taichung, 413008 Taiwan; 4 Taida Horticultural Co., Ltd., Dacun Township, Changhua County 515002 Taiwan; 5 Orchid Research and Development Center, National Cheng Kung University, Tainan, 701 Taiwan

**Keywords:** Flower size, *Fusarium*, *Phalaenopsis* orchids, Resistance, SSR molecular marker, *TCP* orthologous genes, Yellow leaf disease

## Abstract

**Background:**

The orchid industry has seen a recent surge in export values due to the floral morphology and versatile applications of orchids in various markets for medicinal, food additive, and cosmetic usages. However, plant-related diseases, including the yellow leaf disease caused by *Fusarium solani*, have caused significant losses in the production value of *Phalaenopsis* (up to 30%).

**Results:**

In this study, 203 *Phalaenopsis* cultivars were collected from 10 local orchid nurseries, and their disease severity index and correlation with flower size were evaluated. Larger flowers had weaker resistance to yellow leaf disease, and smaller flowers had stronger resistance. For the genetic relationship of disease resistance to flower size, the genetic background of all cultivars was assessed using OrchidWiz Orchid Database Software and principal component analysis. In addition, we identified the orthologous genes of *BraTCP4*, namely *PeIN6*, *PeCIN7*, and *PeCIN8*, which are involved in resistance to pathogens, and analyzed their gene expression. The expression of *PeCIN8* was significantly higher in the most resistant cultivars (A7403, A11294, and A2945) relative to the most susceptible cultivars (A10670, A6390, and A10746).

**Conclusions:**

We identified a correlation between flower size and resistance to yellow leaf disease in *Phalaenopsis* orchids. The expression of *PeCIN8* may regulate the two traits in the disease-resistant cultivars. These findings can be applied to *Phalaenopsis* breeding programs to develop resistant cultivars against yellow leaf disease.

**Supplementary Information:**

The online version contains supplementary material available at 10.1186/s12870-023-04567-3.

## Introduction

Orchidaceae is a diverse family of more than 30,000 species with epiphytic and terrestrial growth forms found in every continent. Tropical orchids show higher speciation rates than those in subtropical or temperate areas. Orchids are popular because of their fabulous morphology and bright-colored flowers. They have various uses and have significant potential value for floriculture, medicinal, and food condiments [1, 2]. In 2022, the export rate of orchids reached more than $209 million USD in Taiwan, with *Phalaenopsis* accounting for more than 80% of the total price [3]. *Phalaenopsis* orchids are distributed from southern China to Southeast Asia [4], with two native species in Taiwan, namely *P. equestris* and *P. aphrodite* subsp. *formosana* (hereinafter *P. aphrodite*). However, the production of *Phalaenopsis* orchids faces several threats such as global climate change, greenhouse hardware limitations, and diseases outbreaks, collectively result in an annual reduction of approximately 30% in export output value [5].

In Taiwan, most *Phalaenopsis* orchids are shipped overseas to countries and packed in the thick cardboard boxes without the supply of light and water for months. Occasionally, the orchid plants show yellow leaf disease syndrome, with infection by *Fusarium solani* upon arrival even though they were healthy before packaging. High temperatures, relative humidity, darkness, and lack of water during long-distance transportation to overseas markets increase the likelihood of disease outbreaks [6]. Breeders need to know the potential risk of infection for any kind of orchids and take comprehensive preventive measures against pathogens while upgrading their transport equipment or cultural facilities. Hence, breeding *Phalaenopsis* orchids with elite aesthetic traits as well as resistance to yellow leaf disease is the primary goal for orchid nurseries.

Yellow leaf disease, caused by the *F. solani* fungal pathogen, is a widespread concern and poses a significant threat to ornamental cash crops such as *Phalaenopsis* spp. [6]. *Fusarium* produces sickle-shaped conidia, which can remain in cultivation materials as chlamydospores and invade the host plant via the emergence of roots or wounds [7]. Symptoms of infection include root decay, black lesions on the abbreviated stem, leaf yellowing, and defoliation. Orange-red perithecia can appear on the lesion site of *Phalaenopsis* orchids and release ascospores to infect adjacent plants when mature [8].

Flower size is a crucial ecological trait that affects mating systems and reproductive success [9, 10]. It can attract specific pollinators, and large flower sizes produce more nectar rewards [11], thus leading to increased pollinator visit rates. The variation in floral morphology affects reproductive isolation more than flower color, given the unique pollination mechanism in Orchidaceae [12].

The *TCP* family of transcription factors was named after members of *TEOSINTE BRANCHED1* (*TB1*) in maize, *CYCLOIDEA* (*CYC*) in snapdragon, and *Proliferating Cell Factor* (*PCF*) in rice [13]. *TCP* family genes control flower development, leaf senescence, and seed germination by regulating cell differentiation and proliferation for plant development [14–16]. The *TCP* family comprises classes I and II genes, with the latter further divided into *CINCINNATA* (*CIN*) and *CYC/TB1* sub-clades [15, 16]. Class I genes (such as *AtTCP8*, *22*, *23*) promote cell proliferation and leaf size in *Arabidopsis* [17]. Class II genes regulate axillary meristem development in maize [18]. Previously, 23 *TCP* genes were identified in the whole genome of *P. equestris* involved in the systematic development of different organs [15].

However, some *TCP* genes have negative effects on plant development. For instance, *TCP4* suppresses petal growth in *Arabidopsis* by repressing cell proliferation [19]. The flower size can be rescued by downregulating the expression of *TCP4* using *microRNA319* [19]. *PePCF10*, a member of class I *TCP* genes in *Phalaenopsis* orchids, was functionally characterized in transgenic *Arabidopsis* overexpressing *PePCF10* or by fusing it to a repressor SRDX domain. *PePCF10* could regulate cell proliferation and differentiation and thus generate a wrinkled surface and downwardly curled rosette leaves [15].

Previously, we developed an efficient inoculation technique for *F. solani* pathogenic assessment in *Phalaenopsis* orchids [20]. In our study, we evaluated the relationship between flower size and resistance to yellow leaf disease using the developed inoculation technique in 203 *Phalaenopsis* commercial cultivars collected from 10 orchid nurseries from central to southern Taiwan. We also assessed their genetic background and the expression of genes possibly related to yellow leaf disease. We found that large-flower *Phalaenopsis* cultivars were more susceptible to yellow leaf disease, whereas the small-flower cultivars showed stronger resistance. In addition, the significantly higher expression of the gene *PeCIN8* in the most resistant cultivars suggests that it may play a role in regulating both flower size and resistance to yellow leaf disease.

## Results

### Disease severity index and susceptibility rank of 203 *Phalaenopsis* cultivars

We assessed the disease severity index (DSI) of the 203 *Phalaenopsis* cultivars after inoculation of *Fusarium* spores (10^3^ spores/ml) in detached leaves. Inoculating Fusarium in detached leaves results in a better phenotyping approach because it reduces the time and space needed in comparison to inoculating the abbreviated stems.

**Fig. 1:**
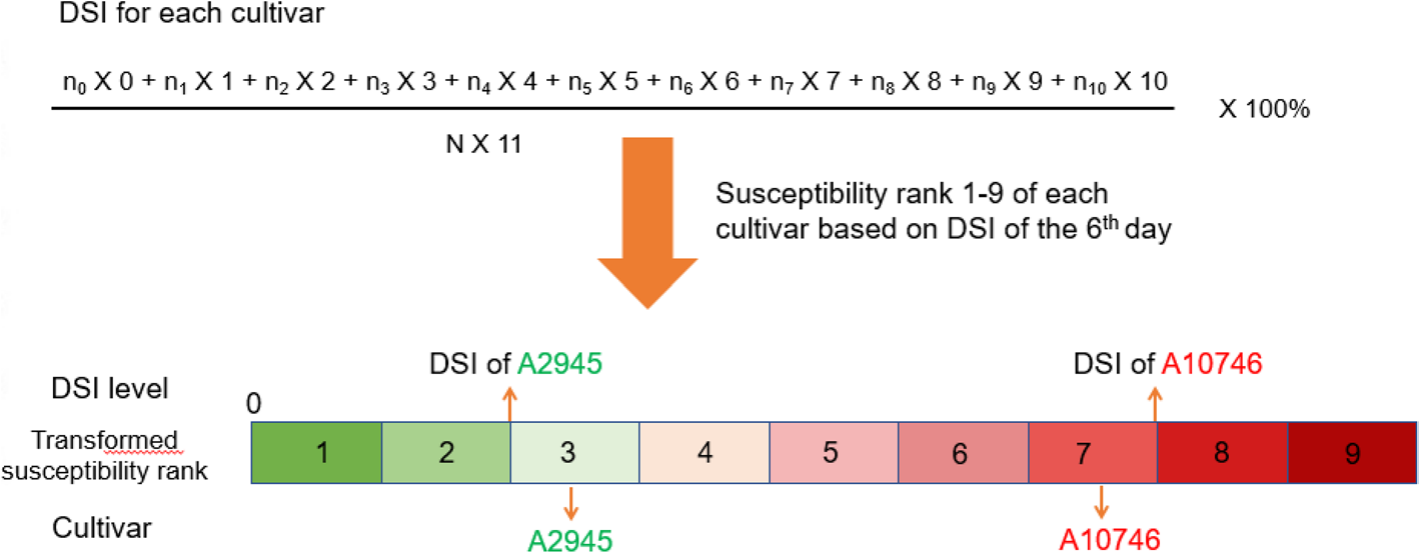
Transformed susceptibility rank using the disease severity index (DSI) of two internal controls. The severity of rank is in gradient color from green to red representing resistant to susceptible. The DSI of A2945 and A10746 is the lowest and highest boundary for rank 3 and 7, respectively. The rank of two controls are always fixed at rank 3 and rank 7, which normalizes all cultivars in various batches of experiments

We recorded the symptom development on infected leaves every day for each plant and photographed them from 0 to 6 days post-inoculation (dpi). A total of 203 cultivars, 4,060 plants, were inoculated from April to July 2020. The DSI was calculated as described [20] (Fig. 1), and the average DSI for the 203 cultivars from 10 orchid nurseries was recorded (Supplementary Fig. S1).

The resistance to *Fusarium* of each *Phalaenopsis* cultivar involves several factors, such as genetic background, developmental stages, and greenhouse management. To avoid differential resistance among individuals of the same cultivar and the fluctuation in DSI for each cultivar, we used two internal controls, resistant (A2945) and susceptible (A10746) cultivars derived from previous experiments [20], in each experiment.

We then transformed the DSI of all cultivars into susceptibility ranks (1–9) by using the DSI on 6 dpi for each experiment. The susceptibility rank is a simple way to inspect the pathogen resistance more intuitively. Furthermore, the susceptibility rank can serve as a valuable reference for breeding. First, we set the scale of total DSI values as nine ranks. The intervals between the DSI of A2945 (rank 3) and A10746 (rank 7) were divided evenly into five sub-intervals. The DSI of A2945 was used as the lower limit of rank three, and that of A10746 as the upper limit of rank seven (Fig. 1). For complete ranks, we used two sub-intervals forward and backward as the most resistant ranks (ranks 1 and 2) and the susceptible ranks (ranks 8 and 9), respectively (Fig. 1). For each individual experiment, the susceptible ranks of the two internal controls were always fixed at ranks three and seven (Fig. 1), which allowed for easily characterizing any cultivars, whether relatively more resistant or more susceptible than controls.

Because *Phalaenopsis* orchids have become a popular ornamental plant in recent years, aesthetic traits such as flower color and flower size have attracted consumers’ attention. Therefore, how to meet the consent of consumers has become the goal of breeders [21]. With the development and progress of biotechnology, many orchid hybrids with fascinating traits have reached global markets [22]. In Taiwan, two native *Phalaenopsis* species, *P. equestris*, and *P. aphrodite*, are widely used as breeding parents. These two species, along with other native species, represent the parental generation that contributes to the genetic diversity of *Phalaenopsis* cultivars. As a result, the average DSI for cultivars greatly differed among 10 nurseries recruited in this study (Supplementary Fig. S1).

**Fig. 2:**
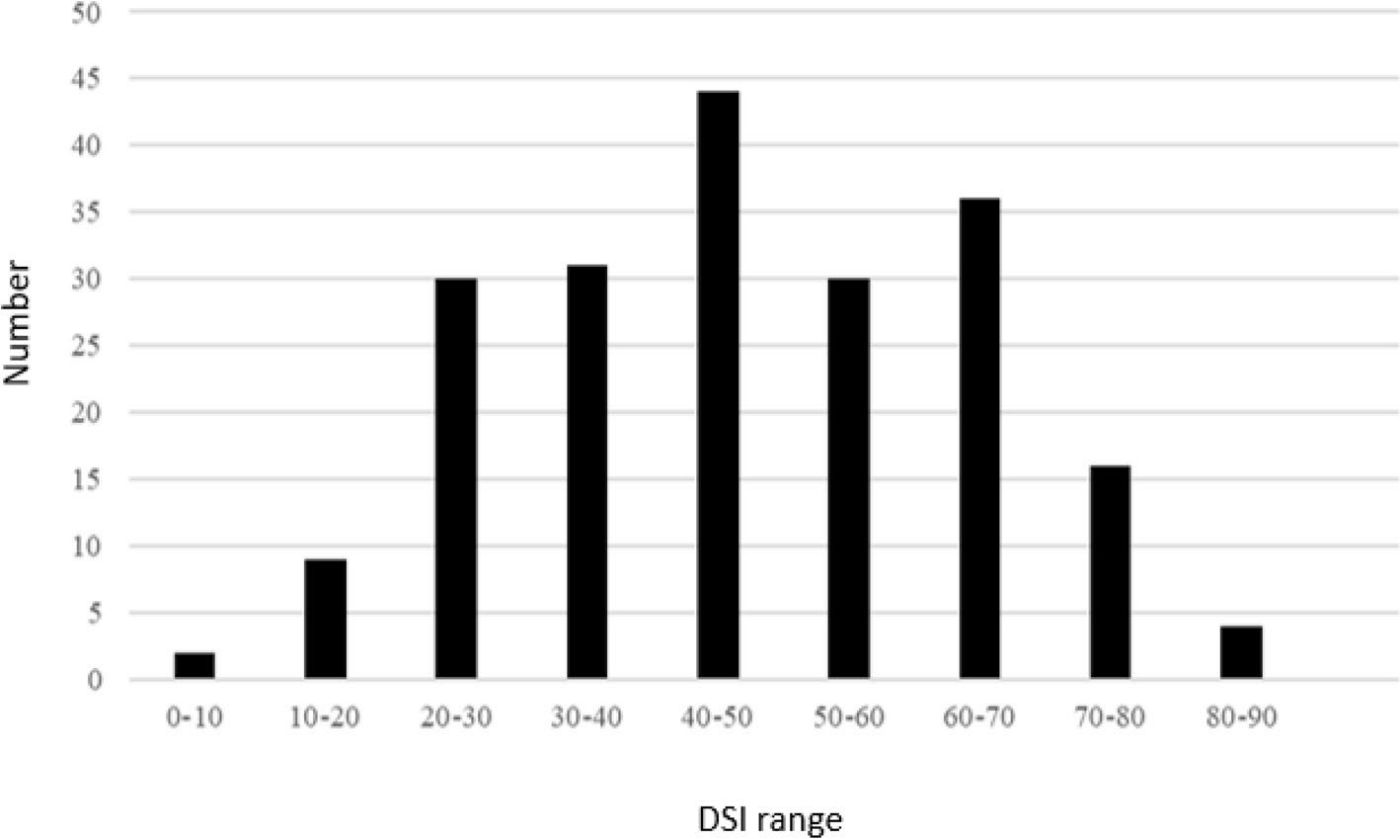
Number of cultivars among all 203 cultivars at different DSI range. The DSI range is separated by intervals of 10 from 0 to 90. A normal distribution is shown, suggesting that the resistance to yellow leaf disease is regulated by quantitative trait loci (QTL)

The DSI values of all 203 cultivars were significantly correlated (two experimental repeats, *R* = 0.64;* p* < 0.0001) (Supplementary Fig. S2), suggesting that reliable and stable data were obtained using the detached leaf method with titers of 1 × 10^3^ spores/ml *Fusarium* spore suspension. In addition, all susceptibility ranks of cultivars showed significantly high correlation between two repeats (*R* = 0.65;* p* < 0.0001) (Supplementary Fig. 3), indicating that the two internal controls selected from the pre-test worked well for normalizing the DSI of various cultivars. This was to avoid the fluctuation of symptom development and resistance performance affected by different environmental factors, such as temperature, light, and humidity during the 4 months of experiments. We recorded the number of cultivars in different DSI ranges and showed a normal distribution from 0 to 90 DSI for all 203 cultivars (Fig. 2), suggesting that the resistance trait to yellow leaf disease may be controlled by multiple genes or quantitative trait loci (QTL).

### Identification of genetic diversity among 203 *Phalaenopsis* cultivars using eight simple sequence repeat (SSR) markers

**Fig. 3:**
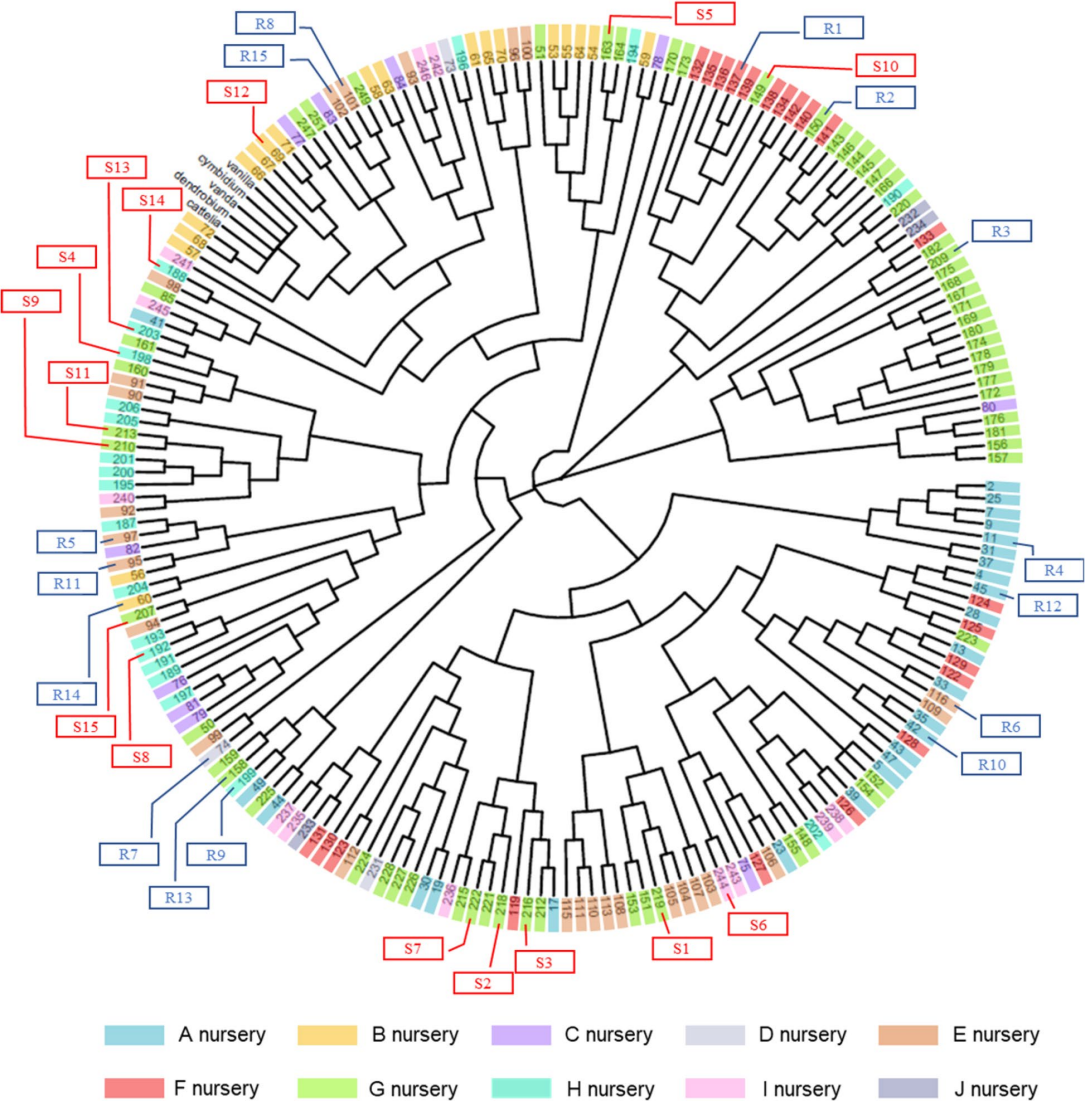
Circular phylogenetic tree for 203 *Phalaenopsis* cultivars and 5 Orchidaceae species. The circular phylogenetic tree was transformed from the linear tree in Supplementary Fig. S5. The numbers in a box with different filled colors represent the sample code corresponding to certain orchid nurseries listed below the tree. The number with the character S or R means the top 15 resistant or susceptible cultivars

To determine the genetic diversity of the 203 *Phalaenopsis*, we resolved the PCR-amplified products of eight SSR markers in 203 cultivars. A total of 203 × 8 = 1624 PCR reactions were performed and resolved in 30 PAGE images (Fig. S4). The SSR data were transformed into a binomial matrix data by visual observation of the banding patterns. A banding on a position with a certain allele length was labeled 1 and otherwise 0 (Supplementary Fig. 5). The phylogenetic tree of 203 *Phalaenopsis* cultivars was established by using NTsys V.2.1 software with the default setting (Supplementary Fig. 6). The phylogenetic tree was further transformed into the unrooted circular tree by using R V.4.1. Each cultivar was labeled by its sample code and marked with different colors corresponding to orchid nurseries A to J. Cultivars within the same orchid nursery were clustered together in the circular phylogenetic tree because of their similar genetic backgrounds (Fig. 3). The top 15 resistant cultivars and top 15 susceptible cultivars according to the DSI list were labeled with blue and red rectangles, respectively, on the periphery of the circular phylogenetic tree (Fig. 3).

### Correlation analysis between DSI and flower size

**Fig. 4:**
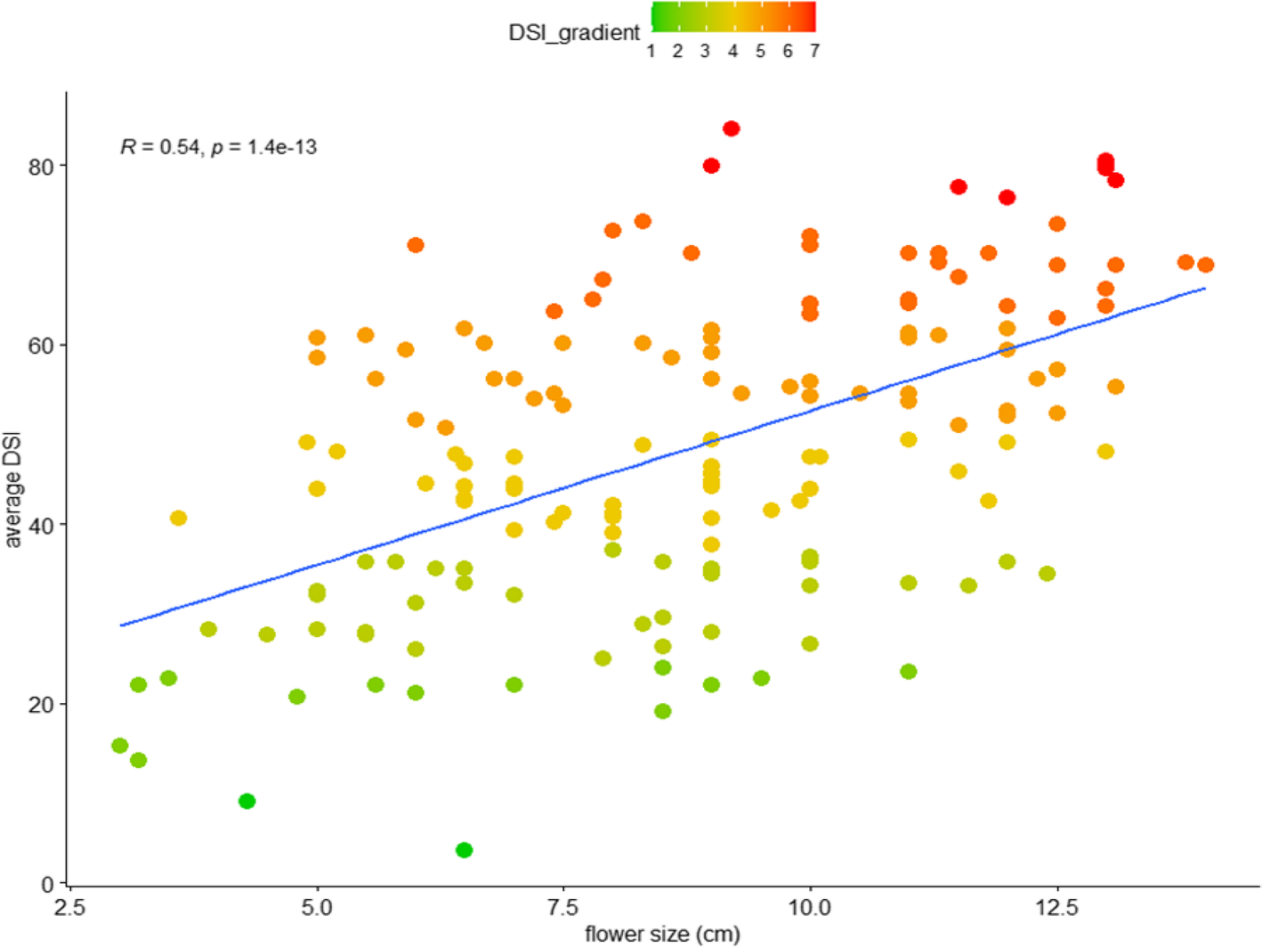
Correlation between average DSI and flower size of 164 cultivars. The x- and y-axes show flower size and average DSI, respectively. The color of the dots indicates the severity of the symptom. The average DSI is in gradient color from green to red representing resistant to susceptible. Pearson’s correlation was conducted, and the correlation coefficient (*R*) is up to 0.54 and *p*-value 1.4*10^–13^

Among the 10 orchid nurseries, seven gave flower size information for a total of 164 cultivars. Correlation analysis of flower size and DSI involved combining flower size data with DSI data for corresponding cultivars. Intriguingly, we found a highly positive correlation between DSI data and flower size (*R* = 0.54, *p* < 0.0001) (Fig. 4), which suggests that larger-flower cultivars were more susceptible to yellow leaf disease and smaller-flower cultivars were more resistant to yellow leaf disease.

### Identification of genetic background affecting DSI and flower size

For genetic background analysis of the *Phalaenopsis* cultivars, we searched OrchidWiz Orchid Database Software (https://www.orchidwiz.com/). There were 12 partial genomes of native *Phalaenopsis* species associated with the parents of the 79 cultivars at different percentages. These species included *P. amabilis*, *P. amboinensis*, *P. aphrodite* subsp. *formosana*, *P. equestris*, *P. gigantea*, *P. hieroglyphica*, *P. pulcherrima*, *P. amabilis* var. *rimestadiana*, *P. sanderiana*, *P. schilleriana*, *P. stuartiana*, and *P. violacea*. To reduce the dimensionality of a large dataset, we transformed a large set of variables into a smaller one that still contained most of the information in the large dataset. We constructed principal component analysis (PCA) plots using percentage data from DSI and flower size.

According to the clustering analysis of genetic background, we found a strong correlation between resistance performance and flower size. The cultivars with large flower size used certain native species as breeding parents, which also increased the susceptibility to *F. solani* infection (Table 1). Similarly, flower size is inversely related to survival rate [27]. Apart from the different greenhouse management and other human factors that may influence the growth vigor of the tested cultivars, the choice of breeding parents was one of the main reasons why orchid nurseries had different ratios of cultivars with various DSI ranges.

**Table 1:** Percentage genomic composition of different parental native *Phalaenopsis* species for top five resistant and susceptible cultivars

**Type**	**Top 5**	***P. equestris***	***P. pulcherrima***	***P. stuartiana***	***P. amabilis***	***P. aphrodite***	***P. amabilis* var*.* rimestadiana**
R	1	22.6	0	3.8	12.6	7.3	13.1
	2	50	25	0	0	0	0
	3	29.9	0	7	9.2	6	10.8
	4	50	0	0.5	18	5.4	10.2
	5	31.9	0.1	14.7	8.2	3.5	7.5
S	1	0	0	0.6	40.1	15.3	41.4
	2	1.1	0.4	1.4	34.8	13.4	32.4
	3	0	0	0.6	40.1	15.3	41.4
	4	3.4	1.5	1.8	31.7	11.2	23.2
	5	3.6	1.1	1.4	29.8	12.1	24.5

**Fig. 5:**
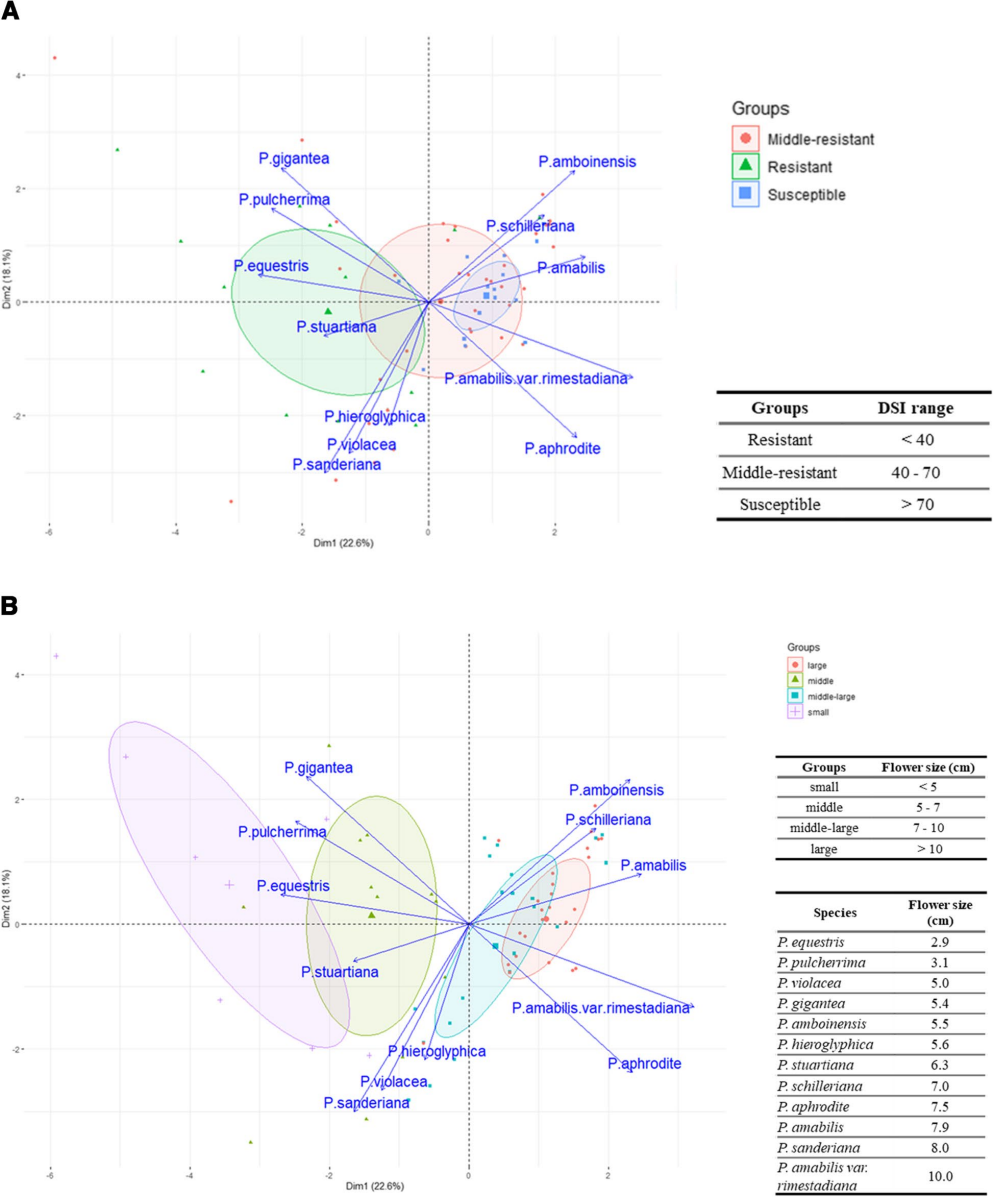
Principal component analysis (PCA) of 79 *Phalaenopsis* cultivars based on the performance of resistance to yellow leaf disease and flower size. **A** The PCA of 79 *Phalaenopsis* cultivars is based on percentage of parenthood. The DSI range of each group is defined (resistant: < 40; middle-resistant: 40–70; susceptible: > 70). The species name in blue color represents the native *Phalaenopsis* species involved in the genome of these cultivars. The blue arrow indicates the influence of these native species in a single dimension and the length of the arrow is positively correlated with influence. **B** The PCA of 79 *Phalaenopsis* cultivars is based on percentage of parenthood. The legend on the bottom left shows 4 groups with different flower diameter. The flower diameter of each group is defined from the table at the bottom middle. The species name in blue color represents the native *Phalaenopsis* species involved in the genome of these cultivars. The flower size of involving native species is shown in the table at the bottom right. The blue arrow indicates the influence of these native species in a single dimension and the length of the arrow is positively correlated with influence

The resistant group was separated from the susceptible one in the PCA results of resistance to yellow leaf data (Fig. 5A, green vs blue color). Several native species such as *P. equestris* and *P. pulcherrima* are used as breeding parents for improving resistance performance. In contrast, species such as *P. amabilis* var. *rimestadiana* and *P. aphrodite* may increase cultivars with susceptibility to *F. solani*.

In addition, different flower size groups were separated (Fig. 5B). Of note, native species such as *P. equestris* and *P. pulcherrima* (2.9–3.1 cm floral diameter) with enhanced resistance to yellow leaf disease have small flowers. In contrast, native species such as *P. amabilis* var. *rimestadiana* and *P. aphrodite* (7.5–7.9 cm floral diameter) have large flowers but are susceptible to yellow leaf disease.

### Identification of *BraTCP4a* orthologous genes in *Phalaenopsis* genome

**Fig. 6:**
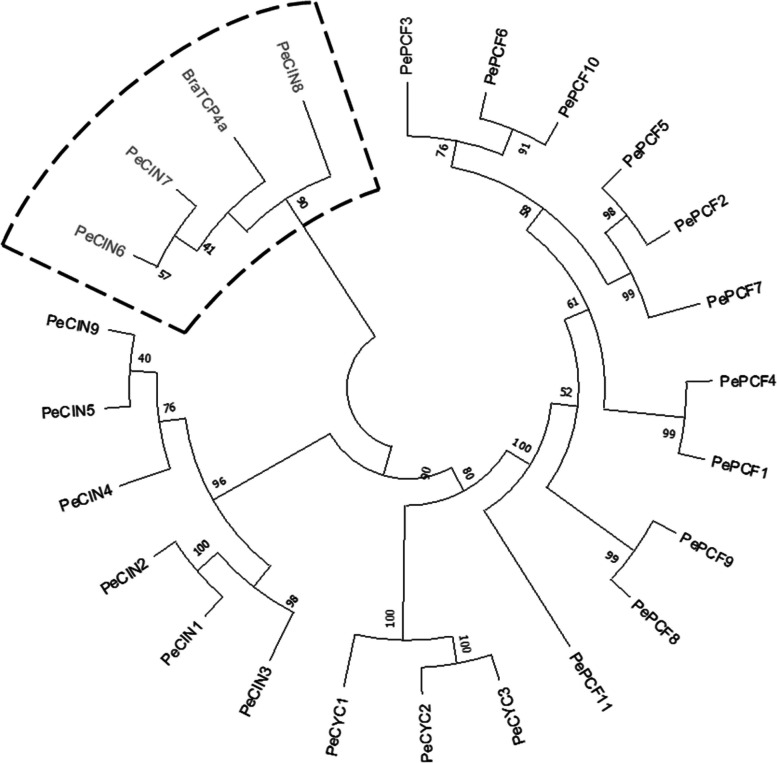
Phylogenetic tree of 23 TCP genes family along with the target gene *BraTCP4a*. The phylogenetic tree is constructed by using alignments in the neighbor-joining method and the parameter JTT model with 1000 bootstrap replicates. Genes in the black-dotted box are clustered in the same clade according to the similarity of amino acid sequence. Therefore, 3 genes (*PeCIN6*, *PeCIN7* and *PeCIN8*) in the dotted box are considered pathogen-related candidate genes and were used for qRT-PCR analysis

Previously, 23 non-redundant genes encoding *TCP* genes were identified in the *P. equestris* genome and provide a great opportunity to identify and characterize TCP transcription factors in orchids [15]. We identified the orthologous genes of *BraTCP4a*, whose expression promotes plant resistance to stem rot disease in *Brassica*, and used phylogenetic analysis with the target gene *BraTCP4a* (Fig. 6). Three orthologous genes, namely *PeCIN6*, *PeCIN7*, and *PeCIN8*, were clustered in the same clade with *BraTCP4a*: both *PeCIN7* and *PeCIN8* are close to each other phylogenetically, and *PeCIN6* is separated (Fig. 6).

### Differential gene expression of *PeCIN* genes for highly resistant and susceptible cultivars

**Fig. 7:**
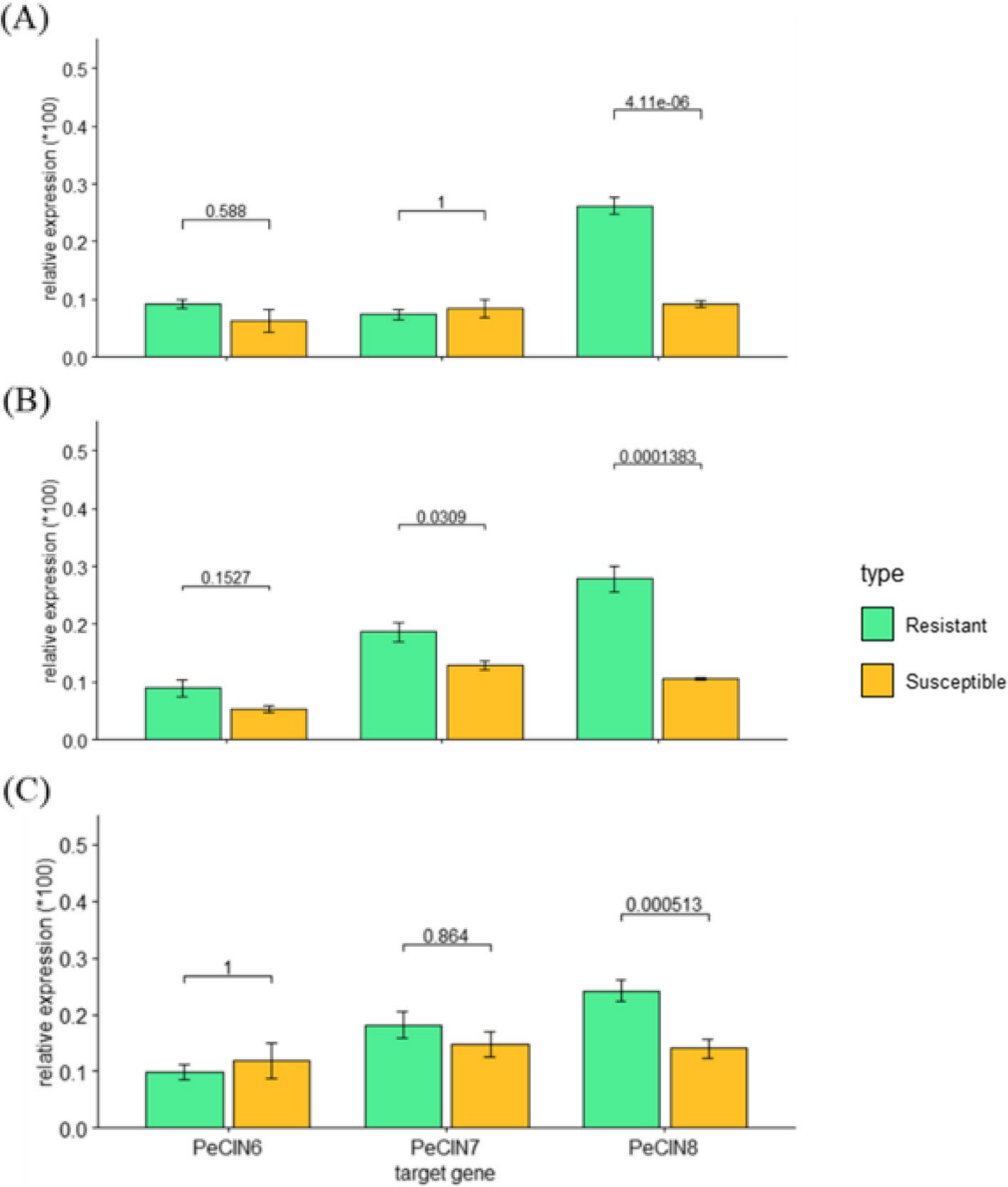
Expression comparison of cultivars in different types for 3 genes. **A** The comparison of resistant (A2945) and susceptible (A10746) internal control. **B** The comparison of the most resistant (A7403) and the most susceptible (A10670) cultivar. **C** The comparison of the top 3 resistant cultivars (A7403, A11294, and A2945) and the top 3 susceptible cultivars (A10670, A6390, and A10746). The x- and y-axes represent different genes and 100-fold relative expression to *Actin1*, respectively. The data are mean ± SE for the cultivars with the same type for each gene and *p-*value across two different types for each gene

We then used qRT-PCR analysis to determine the expression of these three orthologous genes among the *Phalaenopsis* cultivars resistant and susceptible to yellow leaf disease. First, we compared the internal control of the resistant (A2945) and susceptible (A10746) cultivars for expression of the three genes (Fig. 7). The expression of *PeCIN8* in A2945 was significantly higher than that in A10746 (*p* < 0.0001) (Fig. 7A), whereas we found no significant differential expression for both *PeCIN6* and *PeCIN7*. Next, we performed gene expression comparison of three genes in the most resistant (A7403) and the most susceptible (A10670) cultivars (Fig. 7B). Both *PeCIN7* and *PeCIN8* had significantly higher expression in A7403 than A10670 (*p* < 0.05 and *p* < 0.0001) (Fig. 7B and C), with no differential expression of *PeCIN6* between the most resistant and the most susceptible cultivars. Further comparison of the top three resistant (A7403, A11294, and A2945) and top three susceptible (A10670, A6390, and A10746) cultivars showed a significantly differential expression in *PeCIN8* (*p* < 0.001), with no significant differences were detected for both *PeCIN6* and *PeCIN7* (Fig. 7C).

## Discussion

### Genetic diversity of *Phalaenopsis* cultivars

*Phalaenopsis* orchids have huge popularity as ornamental plants worldwide. Hybridization in the breeding program of *Phalaenopsis* is a reliable technique for breeding of elite cultivars with appealing blends of spike length, bud number, flower color and type, floral scent, and compactness to satisfy consumer preferences [2]. Because of advances in biotechnology, many orchid hybrids with attractive traits have been sent to global markets [21]. The two native *Phalaenopsis* species, namely *P. equestris* and *P. aphrodite*, are extensively used as breeding parents in *Phalaenospsis* breeding programs. Alongside other native *Phalaenospsis* species, these two species contribute to the genetic diversity of *Phalaenopsis* cultivars [22]. As a result, the average DSI for cultivars considerably varied among the 10 nurseries recruited for this study (Supplementary Fig. S1).

In this research, the genetic background of *Phalaenopsis* cultivars determined the resistance to yellow leaf disease. By using the OrchidWiz Orchid Database Software, we identified 12 native species as involved in the genetic backgrounds of 79 cultivars. Further analysis using the native genetic background composition percentage revealed distinct clustering groups for different ranges of DSI (Fig. 5A) and flower size (Fig. 5B). The performance of these two traits of flower size and the resistance to yellow leaf disease among the 79 cultivars were regulated by various genetic proportions of the 12 native species, which confirms that both traits were controlled by QTL [23, 24]. Of note, for the top 15 resistant or top 15 susceptible cultivars, we did not clearly observe clustering groups for cultivars with similar resistance or susceptibility in the phylogenetic tree. For susceptible cultivars, small-scale clustering groups were observed at six and ten o’clock positions. However, we detected groups in the eight o’clock position interspersed by several susceptible cultivars as well as the four o’clock position as resistant cultivars (Fig. 3). In addition, native species retained the small-flower genotype because of neutral selection in the process of evolution, when physiological and morphological changes may occur in other traits [25].

According to the clustering analysis of the genetic background, we found a strong correlation between resistance performance and flower size. The cultivars with small flower size native species as their breeding parents (e.g. *P. equesris* and *P. pulcherrima*) showed a reduced susceptibility to *F. solani*. In contrast, the cultivars with large flower size native species as their breeding parents (e.g. *P. aphrodite* and *P. amabilis var. rimestadiana*) showed an enhanced susceptibility to *F. solani*. In *Mimulus guttatus* (monkey flower), intrapopulation QTL of flower size, a highly polygenic trait, features antagonistic pleiotropy: alleles that increase flower size reduce viability but increase fecundity [26]. These observations may explain the balance of selection mechanisms in the evolution of ecologically-relevant traits. The choice of breeding parents was one of the main reasons why orchid nurseries had different ratios of cultivars with various DSI ranges (Supplementary Fig. S1).

### Correlation between flower size and disease resistance

Large flower size can offer advantages in terms of fecundity because it can attract a higher number of pollinators, thereby expanding the plant’s population [9, 10]. However, under stressful environmental conditions, large flowers may become vulnerable, and their viability may be traded for fecundity [27]. Alleles that promote large flower size can increase fertility but decrease survival rates, which supports natural selection favoring plants with small-flower genotypes [26, 28]. Furthermore, orchid cultivars harboring genetic backgrounds of *P. equestris* and *P. pulcherrima* showed remarkably enhanced resistance than those without such lineage. This observation suggests that *P. equestris* and *P. pulcherrima* could be valuable genetic resources for the breeding of cultivars with enhanced resistance against yellow leaf disease. In essence, one approach to identify yellow leaf disease-resistant materials entails examining orchid cultivars with large-flower traits while possessing the genetic heritage of *P. equestris* and *P. pulcherrima*, followed by subjecting them to rigorous tests to assess their resistance capabilities against yellow leaf disease.

### Differential expression of *PeCIN* genes for highly resistant and susceptible cultivars

In *Brassica rapa* plants, overexpression of *BraTCP4-1* promoted plant resistance to infection with *Sclerotinia sclerotiorum* [29]. However, overexpression of *BraMIR319a*, with *BraTCP4-1* as its target gene, increased the susceptibility of the plants to *S. sclerotiorum* infection and then reduced the resistance to stem rot disease in *B. rapa* [30]. To discover the causal genes that regulate resistance to yellow leaf disease, we identified the differential gene expressions of three orthologous genes of *BraTCP4-1*, namely *PeCIN6*, *PeCIN7*, and *PeCIN8*. We detected highly significant differential gene expression of *PeCIN8* in the top three resistant cultivars in comparison to the top three susceptible cultivars (Fig. 7C), while *PeCIN7* showed a significant differential gene expression between the most resistant and the most susceptible line (Fig. 7B).

Cultivars with a close genetic background showed a similar expression pattern for each gene, which may be due to the diversity of genetic backgrounds in which functional genes are inherited from certain native species [22, 30]. In addition, DNA duplication or autopolyploid during meiosis may cause the primers of *TCP* genes off-targeting to express gene sequences with distinct functions [31] or intraspecific gene duplication that drives the *TCP* genes as resident genes for other fundamentally biological functions [32]. Alternatively, other genes may be involved in the resistance to yellow leaf disease in addition to *TCP4* orthologous genes in *Phalaenopsis* orchids.

Although we found that *P. equestris* and *P. pulcherrima* may contribute to resistance against yellow leaf disease, we did not obtain the markers that could be applied to screening in the breeding program. A viable solution involves generating a population crossing *P. equestris* and *P. pulcherrima* with other cultivars exhibiting the large-flower trait. Subsequently, the identification of resistant QTL within this population will allow the identification of markers associated with yellow leaf disease resistance. This comprehensive strategy holds considerable promise in advancing the selection and breeding of *Phalaenopsis* cultivars with enhanced resilience to yellow leaf disease.

The *TCP* gene family, responsible for regulating cell proliferation and cell expansion and then affecting flower development, involves the promotion of biosynthesis and signaling of salicylic acid, jasmonic acid, and abscisic acid under stressful conditions as well [18]. In addition, the *TCP* gene family can trigger the plant-pathogen interaction network and induce the nucleotide-binding domain leucine-rich repeat protein, which increases resistance against pathogens in plants [18]. Plants lacking the function of *TCP13*, *TCP14* and *TCP19* become more vulnerable than the wild type in *Arabidopsis* [33]. In addition, *TCP4* can positively promote the expression of LIPOXYGENASE2, encoding an enzyme that is involved in jasmonic acid biosynthesis in *Arabidopsis* [34], indicating that the upregulating *TCP4* can increase the resistance against pathogens. In addition, *BraTCP4-1* could bind to the promoters of *WRKY75*, *WRKY70*, and *WRKY33* and directly activate pathogen-related genes [29].

However, overexpression of *BSR2*, a cytochrome P450 (CYP78A15), conferred resistance against the bacterial pathogen *Pseudomonas syringa*e pv. tomato DC3000 (PstDC3000) and the fungal pathogens *Colletotrichum higginsianum* and *Rhizoctonia solani* in *Arabidopsis thaliana*. In addition, *BSR2*-overexpressing plants showed enlarged flowers with enlarged floral organs because of the expansion of cells [35]. Thus, different mechanisms may lead to disease resistance and be accompanied with either reduced or enlarged flower size.

## Conclusion

We have discovered a correlation between the flower size and resistance against the yellow leaf disease in *Phalaenopsis* orchids. In addition, we identified the potential role of *PeCIN8* in regulating the flower size trait in highly resistant cultivars. These results have the potential to improve the *Phalaenopsis* breeding programs, by facilitating the development of resilient cultivars that can effectively reduce the impact of yellow leaf disease.

## Materials and methods

### Plant materials of *Phalaenopsis* cultivars

A total of 203 *Phalaenopsis* cultivars/species from 10 local orchid nurseries were collected from central to southern Taiwan. These included 197 *Phalaenopsis* commercial cultivars and 6 native species including *P. aphrodite*, *P. bellina*, *P. equestris*, *P. hieroglyphica*, *P. stuartiana*, and *P. tetraspi*. All plant materials were maintained in 2.5-inch pots for 2–3 months after transfer from glass tissue culture bottles. For each cultivar or native species, 20 plantlets with an average of five to six expanded leaves were used. In each experiment, 10 plantlets were used, with seven plants infected with *Fusarium* and three plants mock-infected with ddH_2_O as negative controls. The second experiment was performed the week after the first experiment. The plant numbers used in this experiment included 4060 (203*10*2) plants for assessing resistance to yellow leaf disease. Plants were well watered and grown at 26℃-29℃ and approximately 70% humidity in various orchid nurseries before they were delivered to National Cheng Kung University (NCKU, Tainan, Taiwan). After arrival, all plant materials were kept under constant temperature at 27 °C to ensure that all plants were maintained at a healthy status before experiments. All plant materials were collected according to institutional guidelines. All *Phalaenopsis* cultivars were commercially available. The authenticity of the plant materials was verified by each orchid nursery owners. A voucher specimen of these materials has been deposited in a publicly available herbarium at NCKU with the voucher deposition numbers of NCKU-LS-1024 ~ 1227.

### Inoculum on detached leaves and identification of symptoms of yellow leaf disease

The inoculum, TJP-2178-10 isolate of *F. solani*, is a fungal pathogen causing yellow leaf disease on orchids. The infection process was as previously described [20]. For orchids infected by *F. solani*, the nutrient substance transportation is cut off and leads to leaf yellowing or even defoliation. As the *F. solani* matures, the pathogen produces red perithecia on the lesion site, which then ruptures and releases ascospores to infect nearby plants.

### Identification of disease severity and transformation from DSI to susceptibility

To distinguish the degree of pathogen resistance for all tested cultivars, we scaled the symptom on each day after infection (DAI) of detached leaves by ranking the disease severity index (DSI) as described [20] (Fig. 1). To standardize the results from various batches of analysis, two internal controls were added, A2945 and A10746, for relative resistant and relative susceptible cultivars, respectively. These two controls were tested to have stable performance in different spore suspension concentrations to normalize the symptom severity for every cultivar within each batch of experiments [20].

We transferred the DSI of all cultivars into susceptibility ranks (1–9) by using the 6^th^-day data on the detached leaf for each experiment. First, we divided the total DSI value into nine ranks. The intervals between the DSI of A2945 (rank 3) and A10746 (rank 7) were divided evenly into 5 sub-intervals. The DSI of A2945 was used as the lower limit of rank 3 and A10746 as the upper limit of rank 7. For complete ranks, we used two sub-intervals forward and backward as extremely resistant ranks (ranks 1 and 2) and more susceptible ranks (ranks 8 and 9), respectively. The rank of the two controls was always fixed at rank three and rank seven, which allowed for easily understanding whether the tested cultivar was relatively more resistant or more susceptible than the controls (Fig. 1).

### DNA extraction and simple sequence repeat (SSR) amplification of 203 *Phalaenopsis* cultivars

For DNA extraction, the newborn leaf shorter than the 2^nd^ leaf from the top was chosen as previously described [20]. To check the genetic diversity of 203 orchid cultivars, we used phylogenetic analysis with SSR markers [36]. In this research, eight SSR markers were used to identify the genetic relationship by genotyping data analyzed with 5% PAGE. In addition, five species of orchids (*Dendrobium nobile*, *Cymbidium goeringii*, *Cattleya violacea*, *Vanilla andamanica*, and *Vanda coerulea*) were added to the analysis as the outgroup.

### Sample preparation for 8 SSR markers

PCR amplification involved the following program: 5 min at 94 °C; 35 cycles of with denaturation (15 s at 94 °C), annealing (15 s at 60 °C) and extension (30 s at 72 °C), then a final step for 5 min at 72 °C. All PCR products were separated in 1% agarose gel at 100 V for 30 min for a quality check. To resolve to 1-bp resolution, 1624 (203*8 = 1624) PCR products were separated with 5% PAGE. The gel casting apparatus contained two rows and 102 wells/row, which allowed for analyzing the maximum 204 PCR samples at once. Finally, a total of 30 PAGE gels were used to analyze the 1624 PCR products.

### Constructing phylogenetic trees with SSR results

The allele size and haplotype of each cultivar analyzed with SSR markers were determined with a binomial matrix by visual observation for different lengths aligned to the DNA ladder [37]. Once there was banding on a position with a certain allele length, the labeling is 1 and otherwise 0. Data were further converted by using Ntedit software (https://anaconda.org/bioconda/ntedit) into a compatible format that could be analyzed using NTsys V.2.1 and constructing the phylogenetic tree for 203 *Phalaenopsis* cultivars along with 5 other orchid species as outgroups.

### Identification of genetic background influencing resistance and flower size

The information on flower size was available from eight orchid nurseries with 79 cultivars registered at The Royal Horticultural Society (https://www.rhs.org.uk/). The genetic background analysis of the 79 registered cultivars was then analyzed using OrchidWiz Orchid Database Software (https://www.orchidwiz.com/). The percentages of genomic composition of parental native *Phalaenopsis* species varied for each cultivar. Clustering analysis was performed according to different resistance or flower size using principal component analysis (PCA) with R v4.1 [38].

### Cultivar selection for gene expression analysis

To understand the difference in the innate defense mechanism against pathogen infection and the floral development between highly resistant and highly susceptible cultivars, we used gene expression analysis with quantitative real-time RT-PCR (qRT-PCR) for candidate genes. Three highly resistant cultivars (A2945, A7403, and A8640) and three highly susceptible cultivars (A10138, A10670 and A10746) were selected according to the correlation analysis between DSI and flower size. Three technical repeats were performed for each cultivar with three biological repeats. Before the experiment, all plants were kept in the greenhouse at NCKU with constant temperature at 27 ~ 29 °C.

### Identification of *BraTCP4a* homologous genes

The *P. equestris* genome contains 23 non-redundant sequences of the *TCP* family [15]. The primer sequences of *BraTCP4a* were used according to Dong et al*.* [30]. The coding sequence of *BraTCP4a* was obtained from the National Center for Biotechnology Information (https://www.ncbi.nlm.nih.gov/). Multiple amino acid sequence alignment was conducted for all 24 TCP sequences using MEGA X software (https://www.megasoftware.net/) [39]. An unrooted phylogenetic tree was constructed following Lin et al. [15], using alignments with the neighbor-joining method and the parameters JTT model with 1000 bootstrap replicates.

### RNA extraction and qRT-PCR

For RNA extraction, all plant materials were maintained in 2.5-inch pots for 2–3 months before transfer from the environment-controlled greenhouses of the orchid nurseries. At this stage, plants have enough time to adapt to the environment and show normal gene expression after interaction with the environment. Total RNA was extracted from the newborn leaf according to a previously described RNA extraction protocol [40]. All RNA samples were used for synthesis of first-strand cDNA with SuperScrip II Reverse Transcriptase kits (Invitrogen, ThermoFisher Scientific, USA) following the manufacturer’s protocol (https://www.thermofisher.com).

The gene expression of putative yellow leaf disease-related genes, including *PeCIN6*, and *PeCIN7*, *PeCIN8* was evaluated in three most resistant cultivars (A2945, A7403, and A8640) and three most susceptible cultivars (A10138, A10670 and A10746) using qRT-PCR. In addition, *Actin1* was recruited as an internal control with the StepOnePlus System (ThermoFisher Scientific, USA). Analysis of gene expression involved three technical repeats and three biological repeats for each cultivar. For statistics analysis, Student *t* test was performed with nine samples for each cultivar. The clustering analysis for all cultivars involved using one-way ANOVA followed by a Tukey post-hoc test with SPSS v17.0 (Computer and Network Center, NCKU).

### Supplementary Information

**Additional file 1: Supplementary Figure 1.** The average DSI comparison of cultivars from all nurseries. **Supplementary Figure 2.** The distribution of DSI of all cultivars between two repeats. **Supplementary Figure 3.** The distribution of susceptibility ranks from all cultivars between two repeats. **Supplementary Figure 4.** The partial PAGE image reveals the banding pattern of SSR markers. **Supplementary Figure 5.** The flow chart of how the SSR analysis data were transformed into binomial matrix data. **Supplementary Figure 6.** The phylogenetic tree of 203 *Phalaenopsis* cultivars and other 5 orchid species.

## Data Availability

All datasets used and/or analyzed during the current study are available from the corresponding author on reasonable request.

## Abbreviations

DSI: Disease severity index
PAGE: Polyacrylamide gel electrophoresis
PCA: Principal component analysis
SSR: Simple sequence repeat
TCP: TEOSINTE BRANCHED/CYCLOIDEA/PCF
