# Supplementary material for: PeCIN8 expression correlates with flower size and resistance to yellow leaf disease in Phalaenopsis orchids
Source: BMC Plant Biol. 2023 Nov 7;23:545. doi: 10.1186/s12870-023-04567-3 (PMC10629045; doi:10.1186/s12870-023-04567-3)
Supplement: Supplementary file 1 — Additional file 1: Supplementary Figure 1. The average DSI comparison of cultivars from all nurseries. Supplementary Figure 2. The distribution of DSI of all cultivars between two repeats. Supplementary Figure 3. The distribution of susceptibility ranks from all cultivars between two repeats. Supplementary Figure 4. The partial PAGE image reveals the banding pattern of SSR markers. Supplementary Figure 5. The flow chart of how the SSR analysis data were transformed into binomial matrix data. Supplementary Figure 6. The phylogenetic tree of 203 Phalaenopsis cultivars and other 5 orchid species. [file 12870_2023_4567_MOESM1_ESM.pdf]

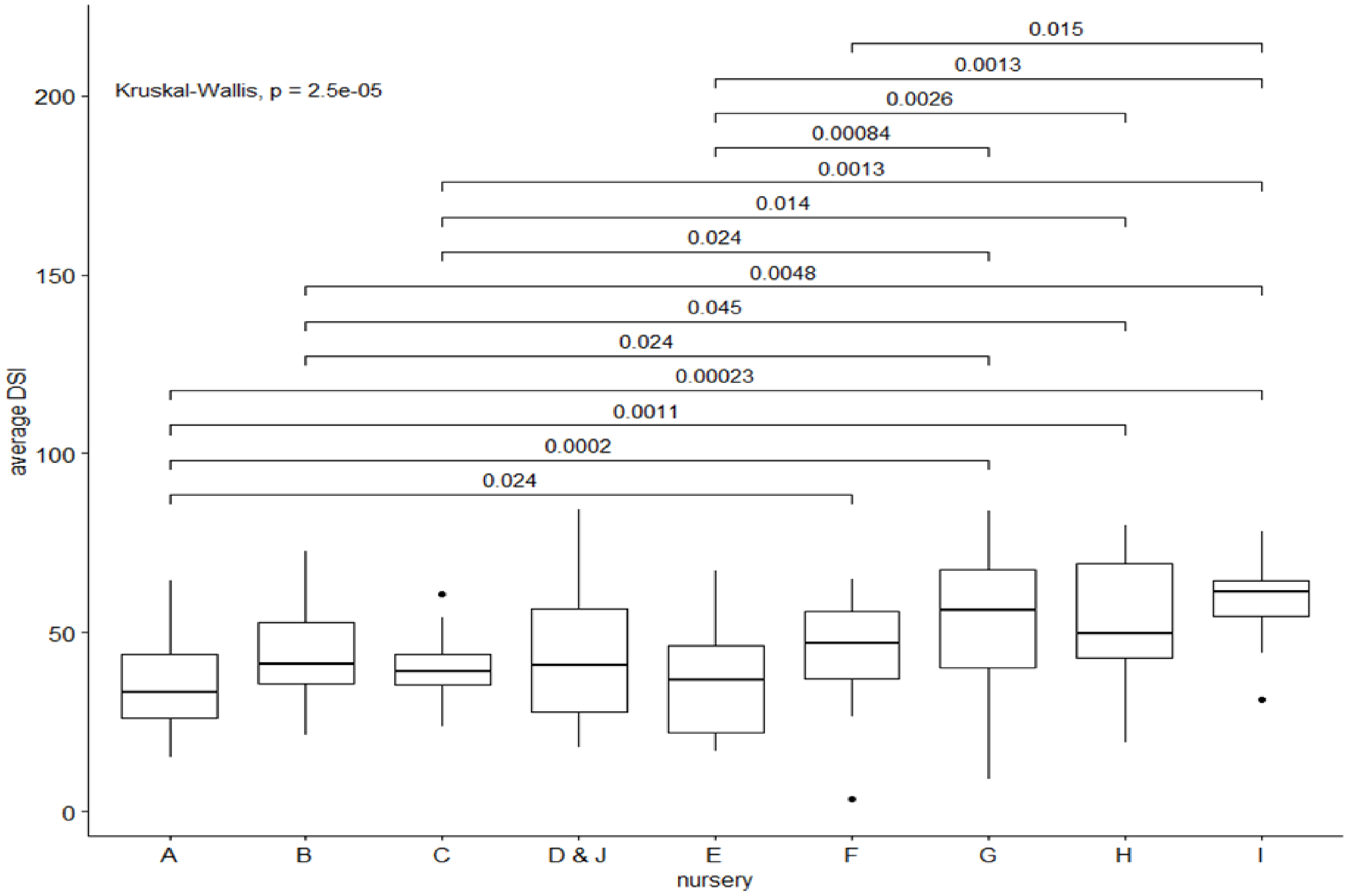

Supplementary Figure 1. The average DSI comparison of cultivars from all nurseries.

The boxplot shows the average DSI comparison of cultivars from all nurseries. The number above the boxes represent the *p*-value of comparison for two corresponding nurseries. Among all nurseries recruited in this research show a significant difference in average DSI ($p < 0.005$).

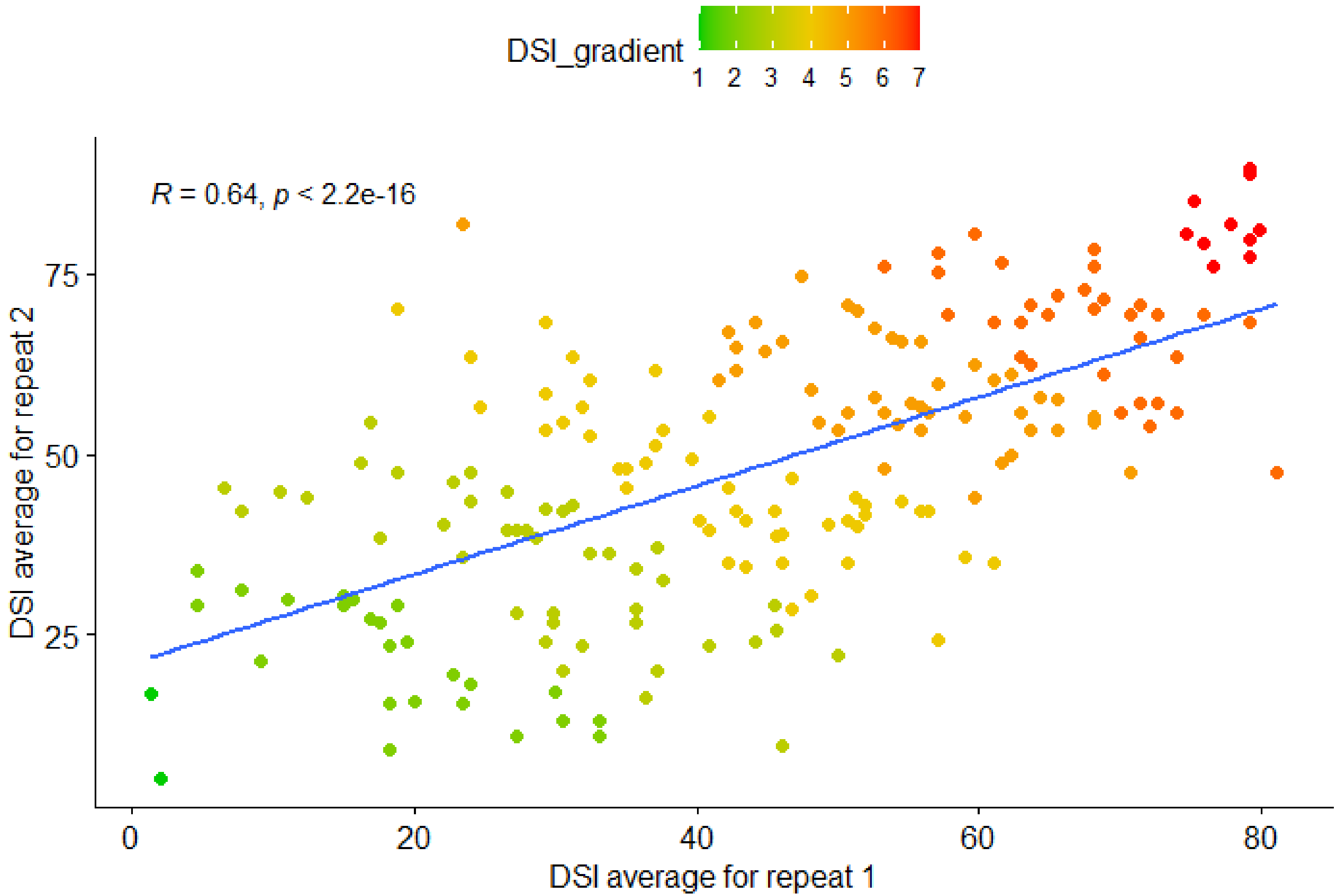

Supplementary Figure 2. The distribution of DSI of all cultivars between two repeats.

The x and y axis show the DSI for each cultivar in repeat 1 and repeat 2, respectively. The color of the dots indicates the severity of the symptom from two repeats. The value of DSI shows in gradient color from green to red represent from resistant toward susceptible. The Pearson's correlation is conducted for two data, and the correlation coefficient (R) is up to 0.64 while the *p* value is less than $2.2*10^{-16}$

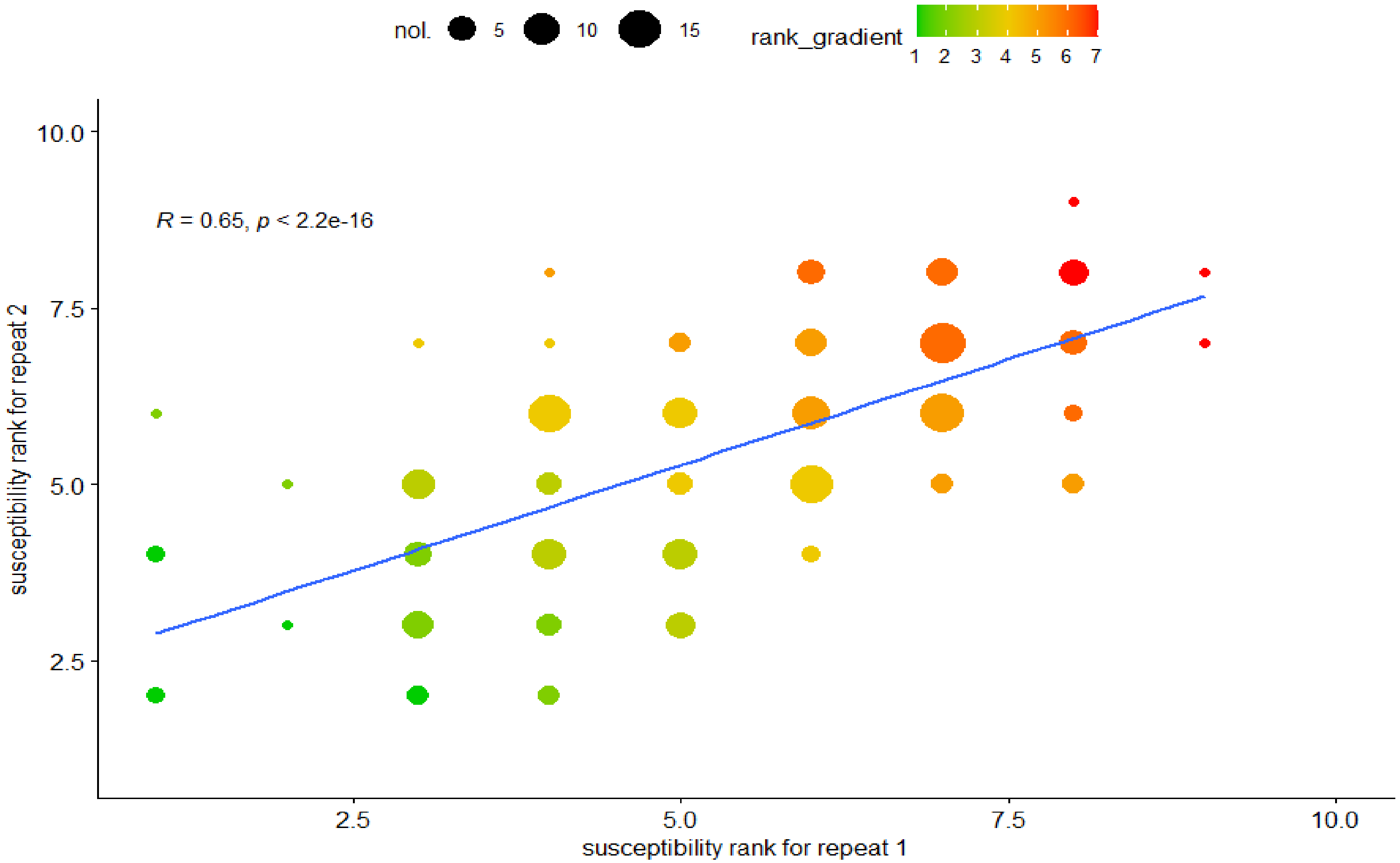

Supplementary Figure 3. The distribution of susceptibility ranks from all cultivars between two repeats.

The x and y axis show the average of susceptibility rank for each cultivar transferred from internal controls in repeat 1 and repeat 2, respectively. The size of dots indicates the number of the cultivars from the same ranking pair. The value of susceptibility rank shows in gradient color from green to red representing from resistant toward susceptible. The Pearson's correlation is conducted for two data, and the correlation coefficient (R) is up to 0.65 while the *p* value is less than $2.2*10^{-16}$

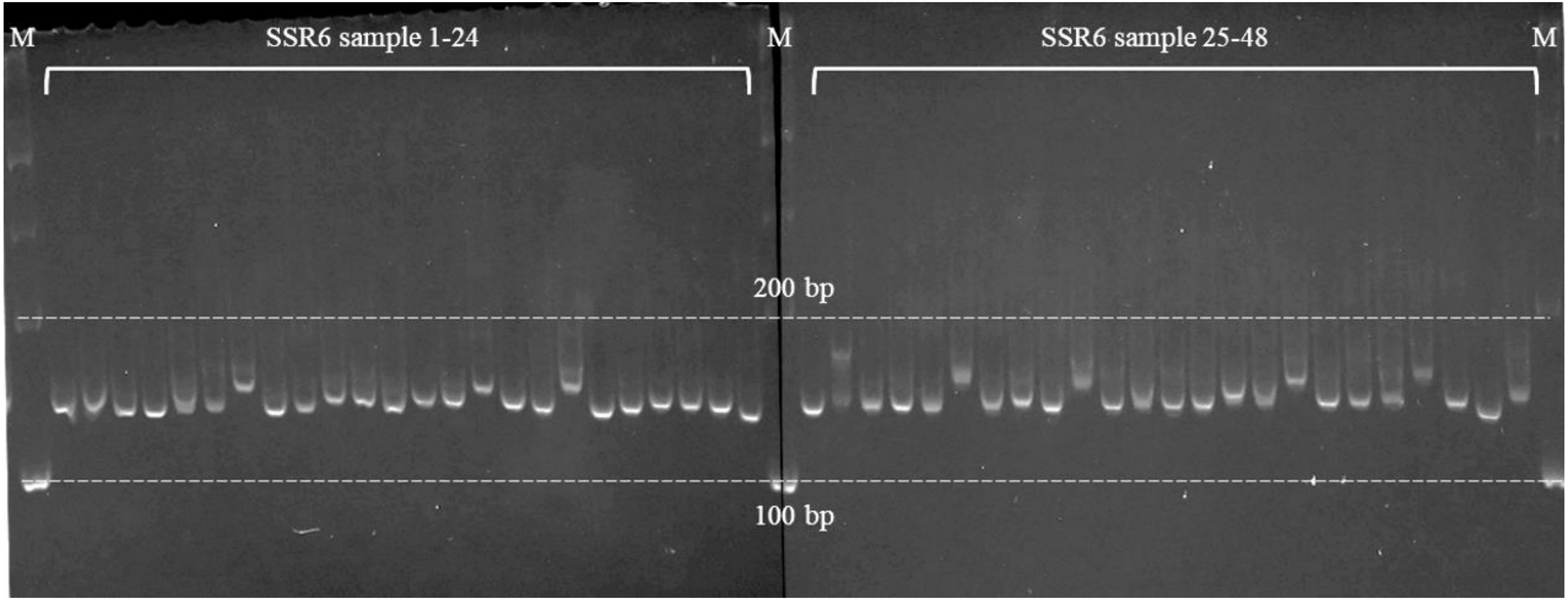

Supplementary Figure 4. The partial PAGE image reveals the banding pattern of SSR markers.

Here only shows SSR6 among 48 of 208 DNA samples, M indicates the 100 bp DNA ladder. The gel images are combined and corrected by the DNA ladder for visual observation for various allele length, the transformed data is shown as binomial matrix listed in Table 3.

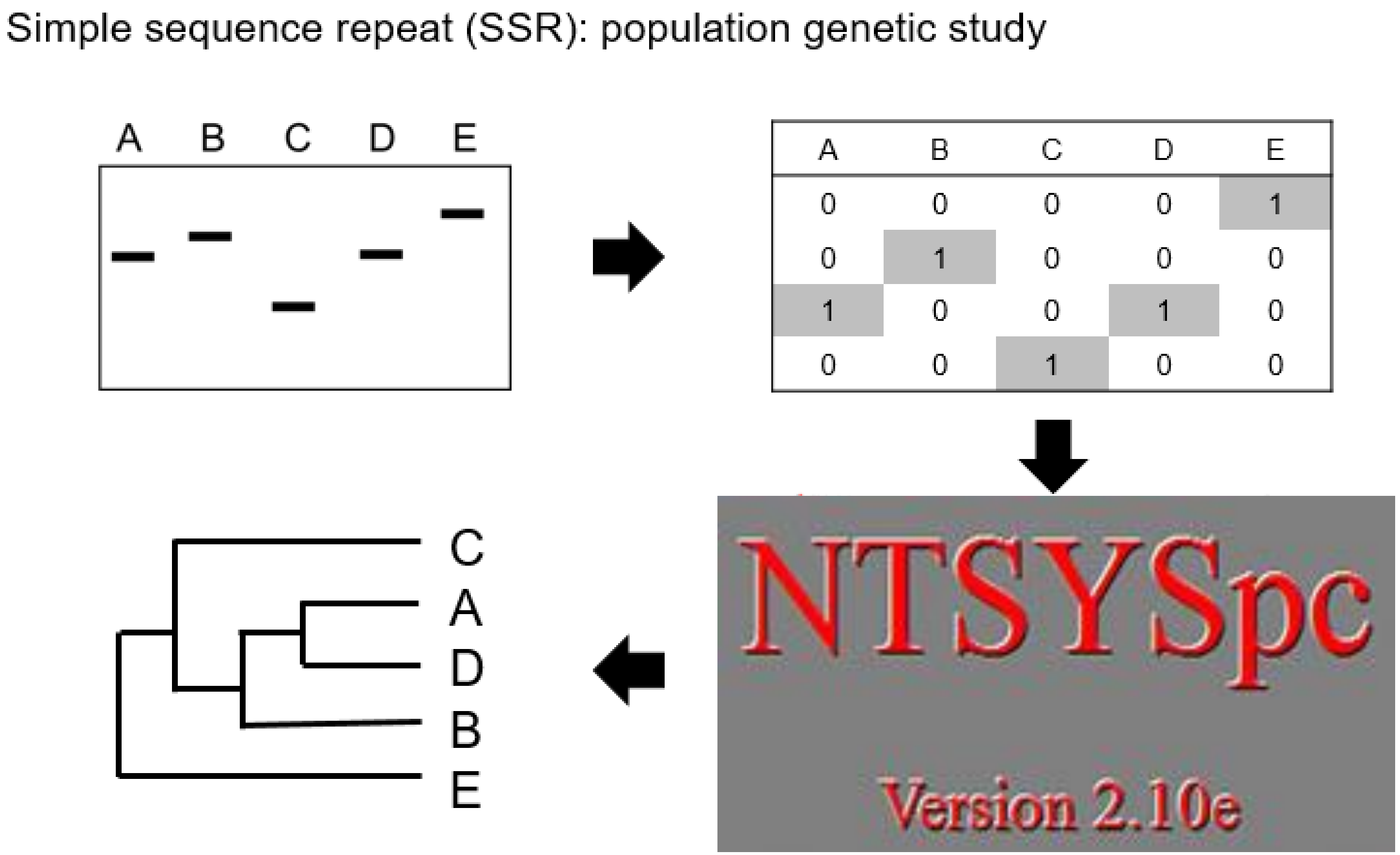

Supplementary Figure 5. The flow chart of how the SSR analysis data were transformed into binomial matrix data.

The SSR analysis data were transformed into binomial matrix data. using visual observation of the banding patterns. Once there was a banding on position with certain allele length would be labeled as 1, if not then labeled as 0 instead

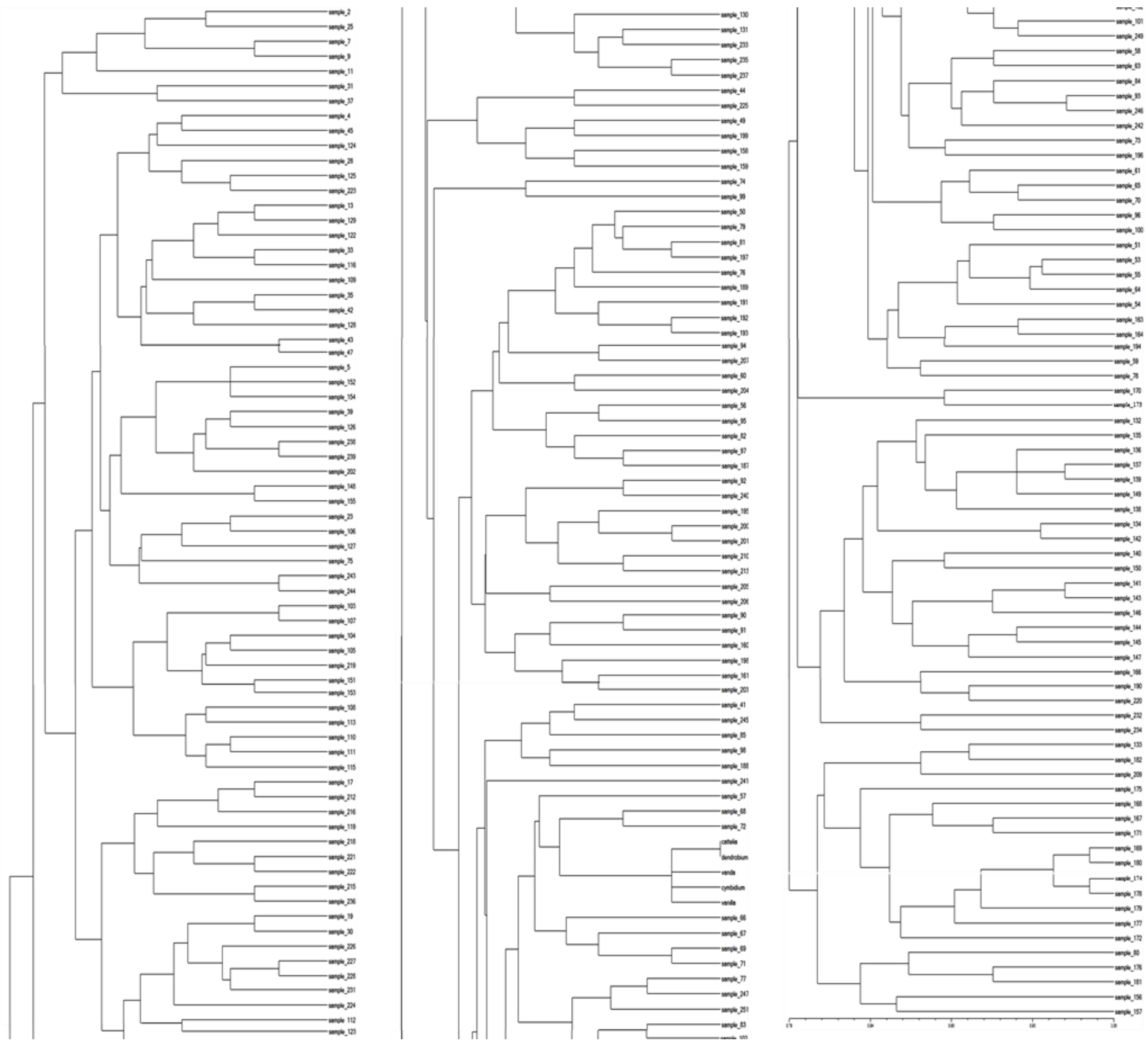

Supplementary Figure 6. The phylogenetic tree of 203 *Phalaenopsis* cultivars and other 5 orchid species.

The phylogenetic tree is constructed according to binomial matrix listed in Table 3 by using the NTsys software (version 2.1). Close genetic background is identified among all cultivars due to the genetic relevance is higher than 0.8 separated by 8 SSR markers.
